# Supplementary material for: In-Depth Analysis of the N-Glycome of Colorectal Cancer Cell Lines
Source: Int J Mol Sci. 2023 Mar 2;24(5):4842. doi: 10.3390/ijms24054842 (PMC10003090; doi:10.3390/ijms24054842)
Supplement: Supplementary file 1 [file ijms-24-04842-s001.zip › Supplementary figures.pdf]

International Journal of *Molecular Sciences*

MDPI

Supplementary Information

In-depth Analysis of the *N*-glycome of Colorectal Cancer Cell Lines

Di Wang [1,†], Valeriia Kuzyk [1,2,†], Katarina Madunić [1,3], Tao Zhang [1], Oleg A. Mayboroda [1], Manfred Wuhrer [1] and Guinevere S. M. Lageveen-Kammeijer [1,4,*]

[1] Center for Proteomics and Metabolomics, Leiden University Medical Center, Postbus 9600, 2300 RC Leiden, The Netherlands
[2] Division of Bioanalytical Chemistry, Vrije Universiteit Amsterdam, 1081 HV Amsterdam, The Netherlands
[3] Department of Cellular and Molecular Medicine, Copenhagen Center for Glycomics, University of Copenhagen, 2200 Copenhagen, Denmark
[4] Analytical Biochemistry, Groningen Research Institute of Pharmacy, University of Groningen, 9700 AD Groningen, The Netherlands
* Correspondence: g.s.m.kammeijer@rug.nl
† These authors contributed equally to this work.

Contents

Supplementary Information Figure S1 2
Supplementary Information Figure S2 3
Supplementary Information Figure S3 4
Supplementary Information Figure S4 5
Supplementary Information Figure S5 14
Supplementary Information Figure S6 15
Supplementary Information Figure S7 16

International Journal of
Molecular Sciences

MDPI

Supplementary Information Figure S1

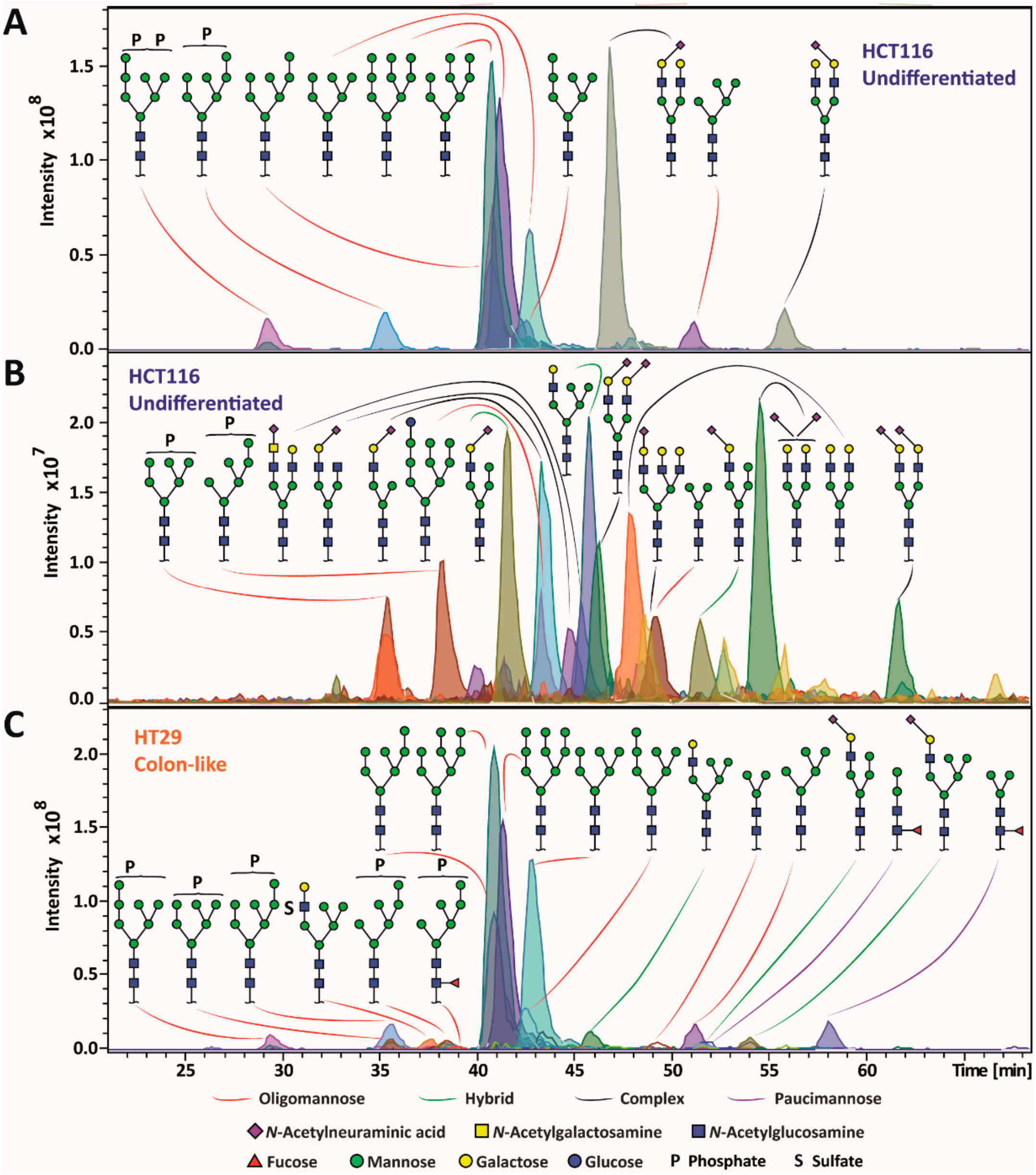

Supplementary Information Figure S1. *N*-glycan profiles of two exemplary CRC cell lines. (**A, B**) The undifferentiated HCT116 cell line shows a higher diversity in glycosylation. (**A**) The 10 most abundant of *N*-glycans are presented whereas (**B**) illustrates the lower abundant *N*-glycans (> 0.5% relative abundance) (**C**) Colon-like cell line HT29 is mainly dominated by oligomannosidic *N*-glycans. Sialic acid tilted to the left and right indicates α2-3 linkage and α2-6 linkage, respectively. Unidentified linkages are indicated with a sialic acid pointing vertically.

International Journal of
Molecular Sciences

MDPI

Supplementary Information Figure S2

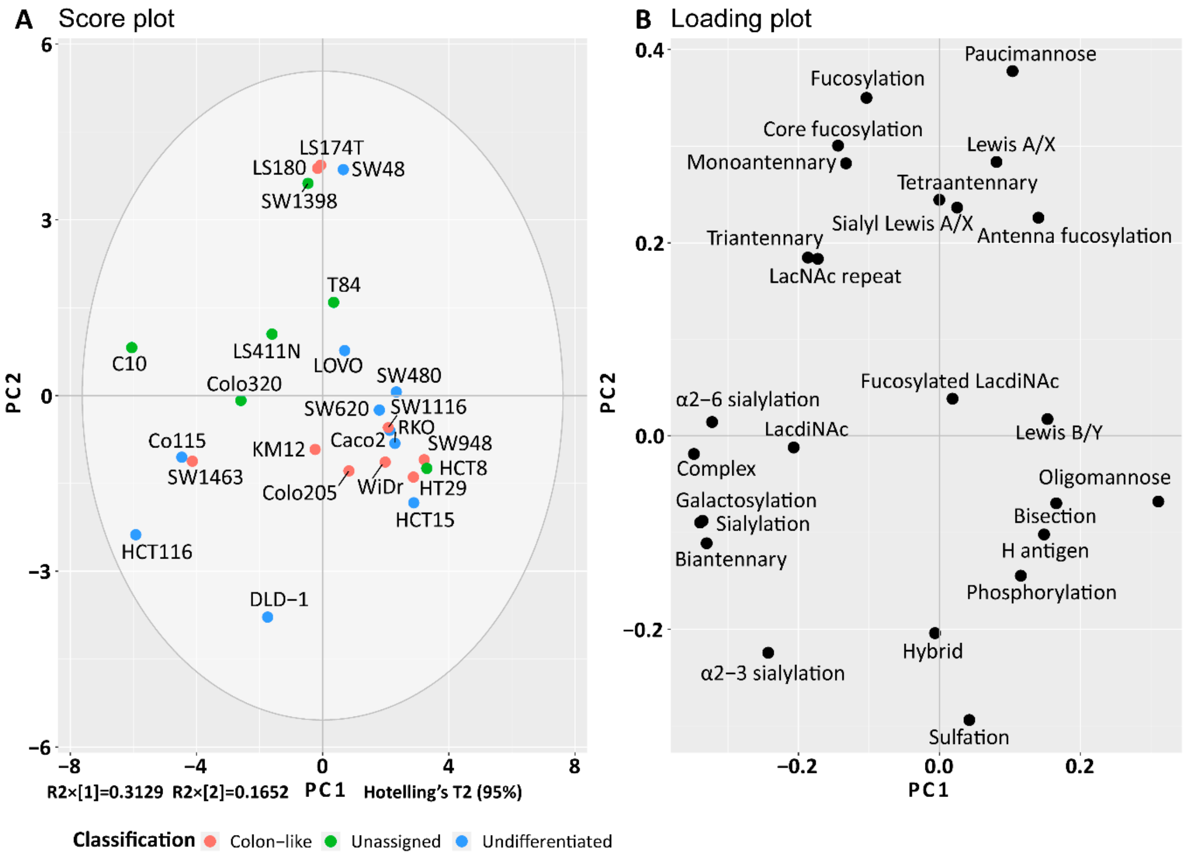

Supplementary Information Figure S2. Principle component analysis (PCA) of *N*-glycosylation features in CRC cell lines. PCA analysis was performed based on the relative quantification of glycosylation features in CRC cell lines. **(A)** Score plot of CRC cell lines colored based on cell line classifications. **(B)** The loading plot indicated the involvement of glycosylation features in the distribution of CRC cell lines in the score plot. The top two PCs hold 46.97% of the variation within the data.

International Journal of *Molecular Sciences*

MDPI

Supplementary Information Figure S3

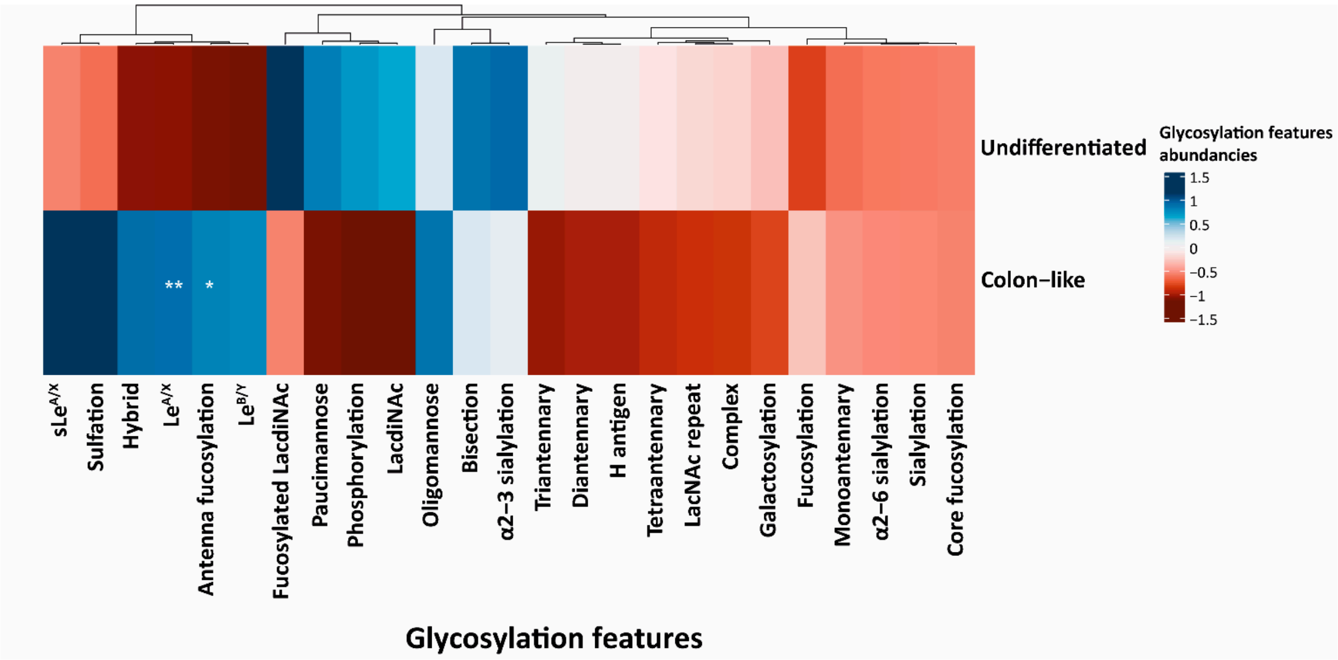

Supplementary Information Figure S3. Heatmap depicting the average relative abundance of glycosylation features (bottom) for CRC cell line classifications (right). The average relative abundance of each glycosylation feature was calculated. The standardization with a mean equal to zero and a standard deviation equal to one was performed. The Wilcoxon—Mann–Whitney non-parametric statistical test was applied to test differences of glycosylation features between colon-like and undifferentiated groups and p-values were corrected by the Benjamini-Hochberg method. Significant values are marked with * ($p \leq 0.05$) and ** ($p \leq 0.01$).

Supplementary Information Figure S4

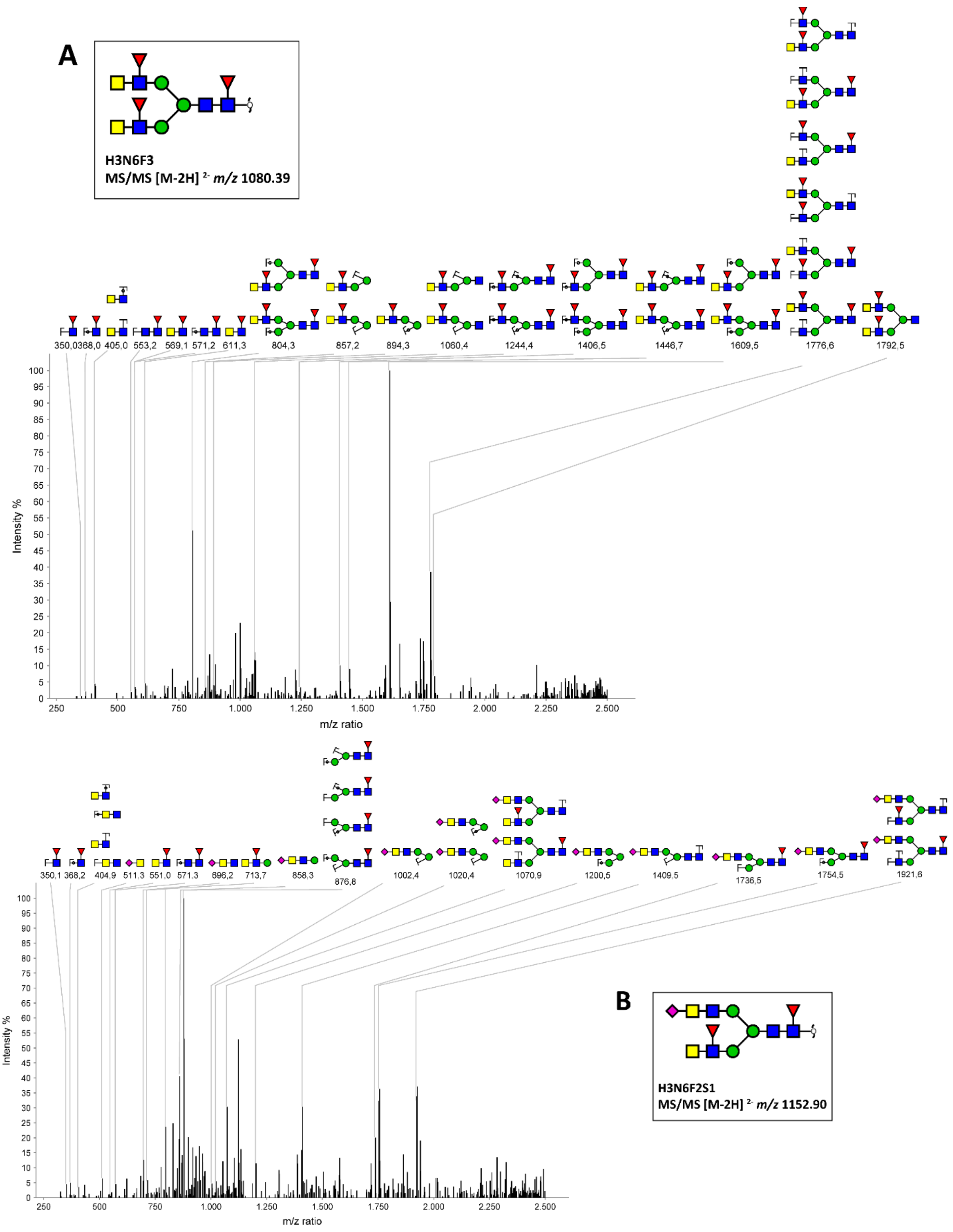

Supplementary Information Figure S4. MS/MS spectra of the selected *N*-glycans: **A)** Fucosylated LacdiNAc carried *N*-glycan with composition H3N6F3; **B)** LacdiNAc carried *N*-glycan with composition H3N6F2S1. Blue square: *N*-acetylglucosamine, green circle: mannose, yellow circle: galactose, red triangle: fucose, purple diamond: *N*-acetylneuraminic acid.

C

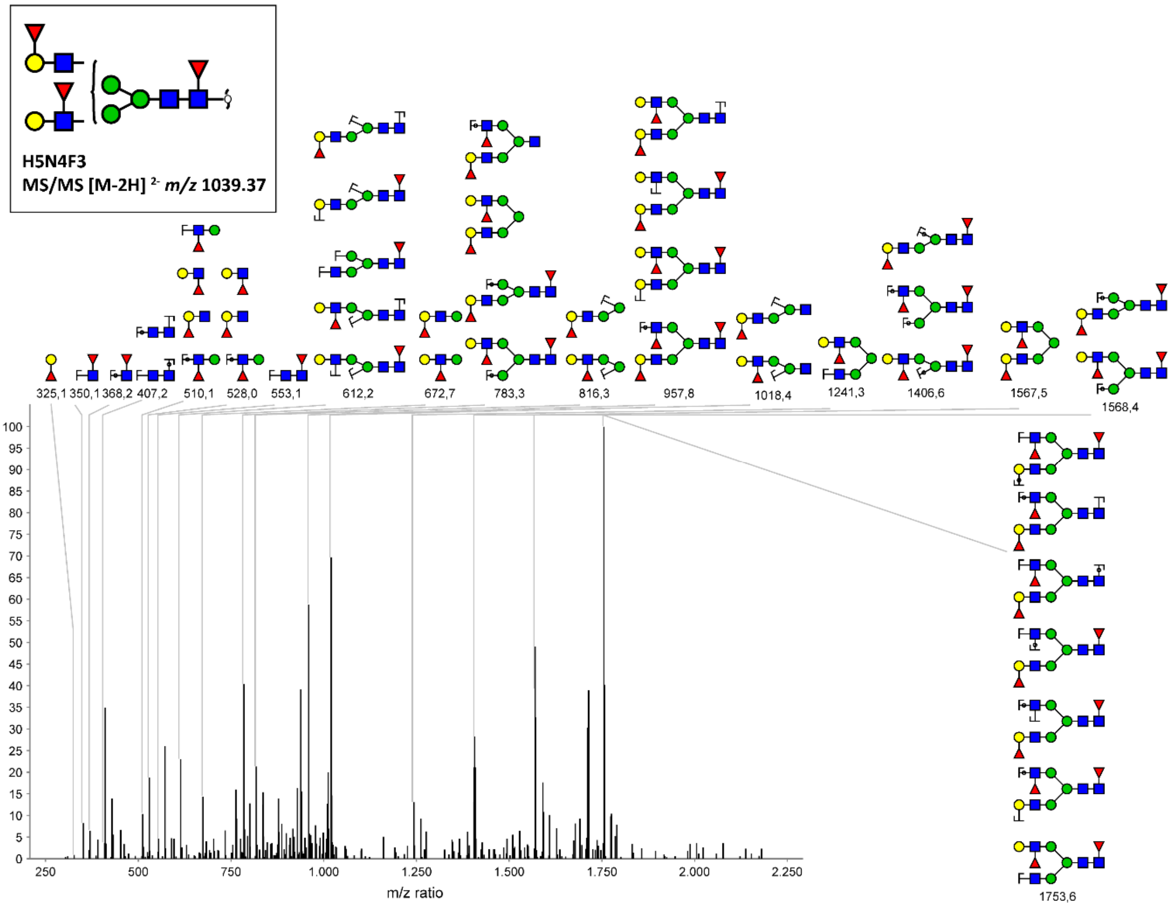

Supplementary Information Figure S4. MS/MS spectra of the selected *N*-glycans (*continued*): C) *N*-glycan carrying H antigen and Lewis antigen with composition H5N4F3. Blue square: *N*-acetylglucosamine, green circle: mannose, yellow circle: galactose, red triangle: fucose, purple diamond: *N*-acetylneuraminic acid.

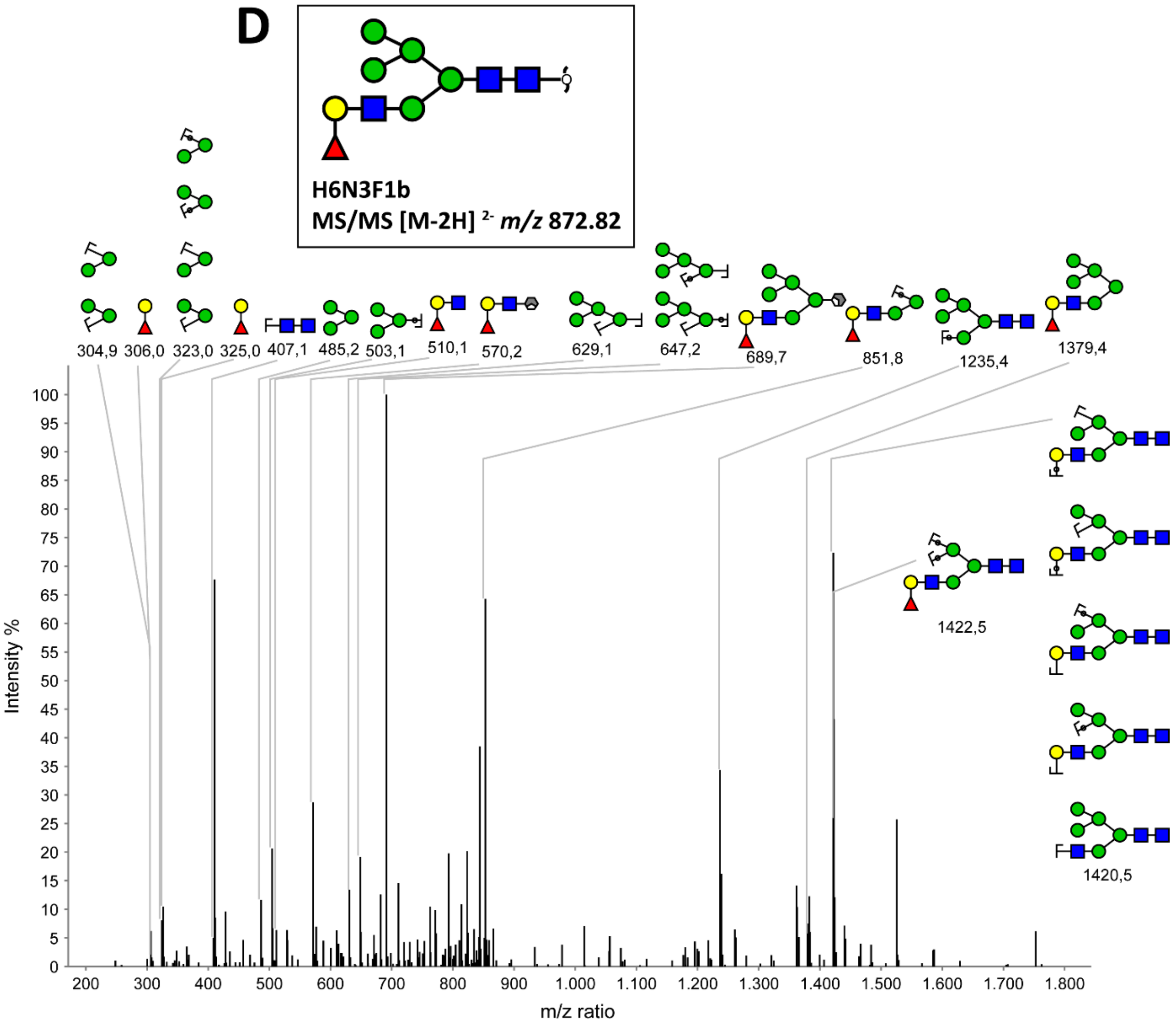

Supplementary Information Figure S4. MS/MS spectra of the selected *N*-glycans (*continued*): D) *N*-glycan carrying H antigen with composition H6N3F1b. Blue square: *N*-acetylglucosamine, green circle: mannose, yellow circle: galactose, red triangle: fucose.

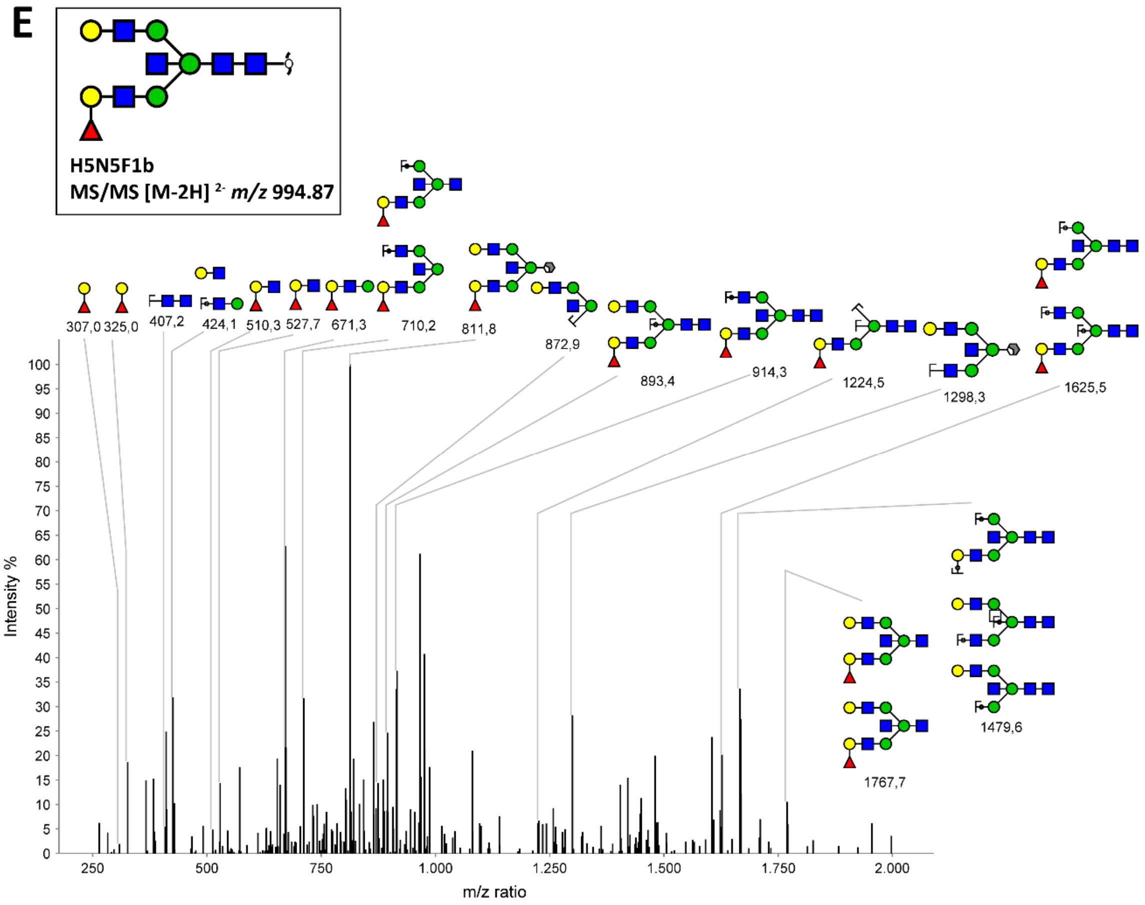

Supplementary Information Figure S4. MS/MS spectra of the selected *N*-glycans (*continued*): E) *N*-glycan carrying H antigen with composition H5N5F1b. Blue square: *N*-acetylglucosamine, green circle: mannose, yellow circle: galactose, red triangle: fucose.

International Journal of
Molecular Sciences

F

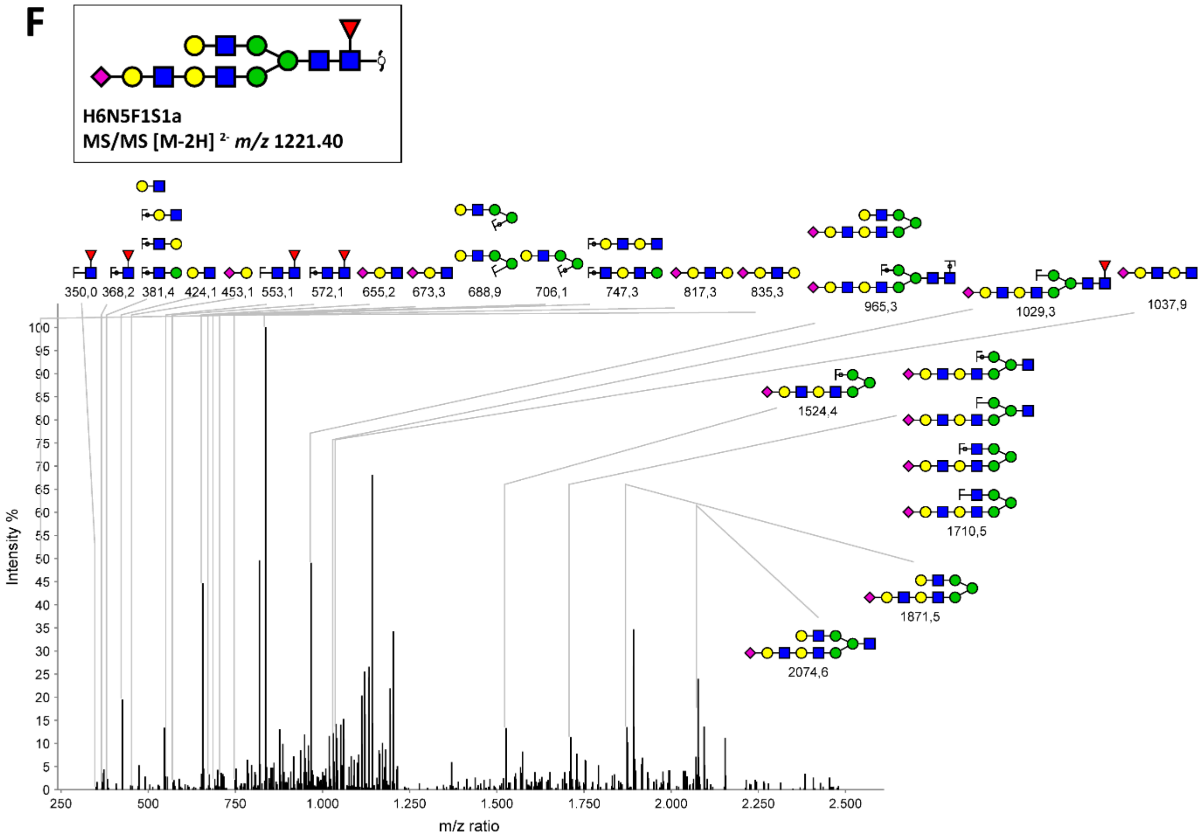

Supplementary Information Figure S4. MS/MS spectra of the selected *N*-glycans (*continued*): F) Repeated LacNAc carried *N*-glycan with composition H6N5F1S1a; Blue square: *N*-acetylglucosamine, green circle: mannose, yellow circle: galactose, red triangle: fucose, purple diamond: *N*-acetylneuraminic acid.

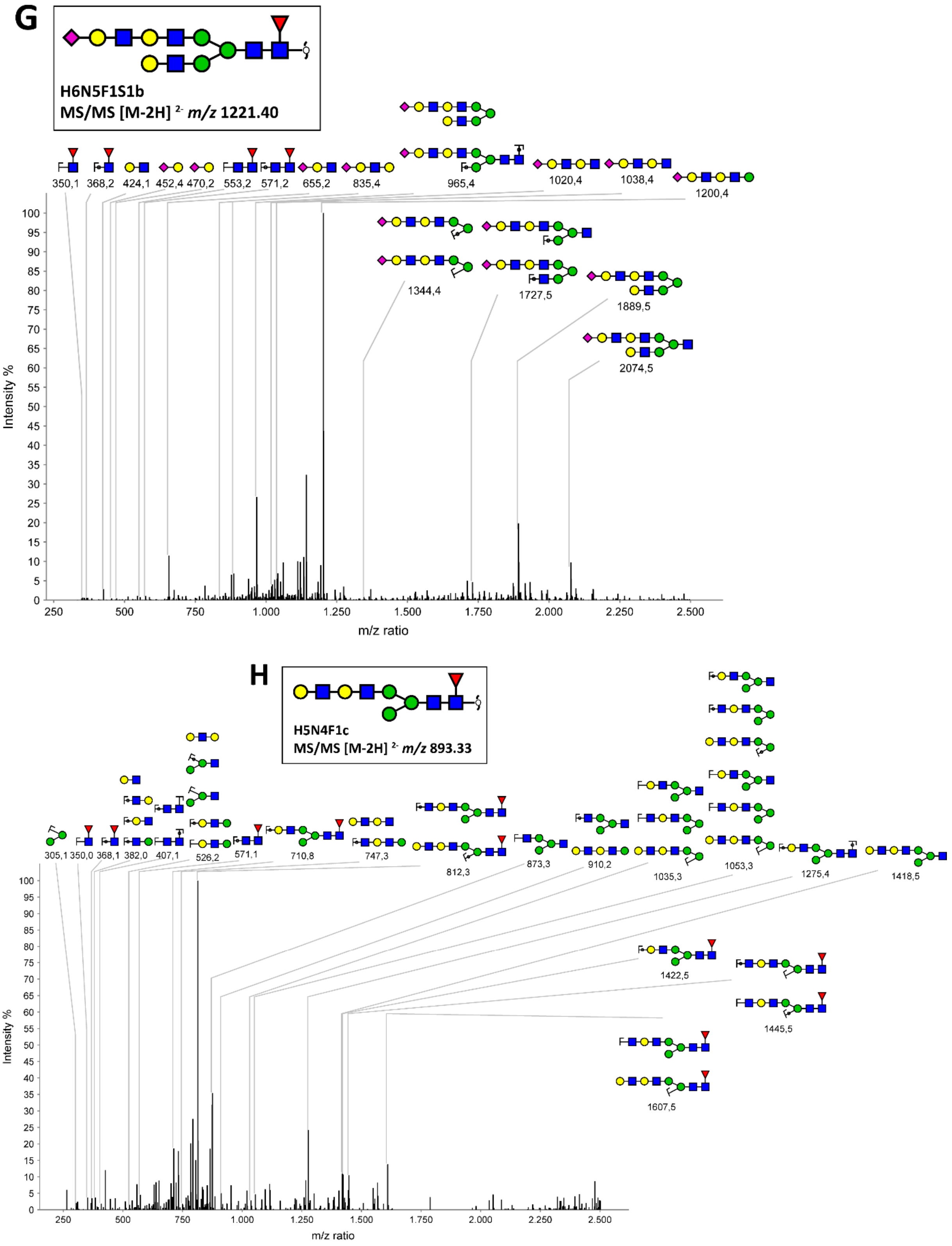

Supplementary Information Figure S4. MS/MS spectra of the selected *N*-glycans(*continued*): G) Repeated LacNAc carried *N*-glycan with composition H6N5F1S1b; **H)** Repeated LacNAc carried *N*-glycan with composition H5N4F1c. Blue square: *N*-acetylglucosamine, green circle: mannose, yellow circle: galactose, red triangle: fucose, purple diamond: *N*-acetylneuraminic acid.

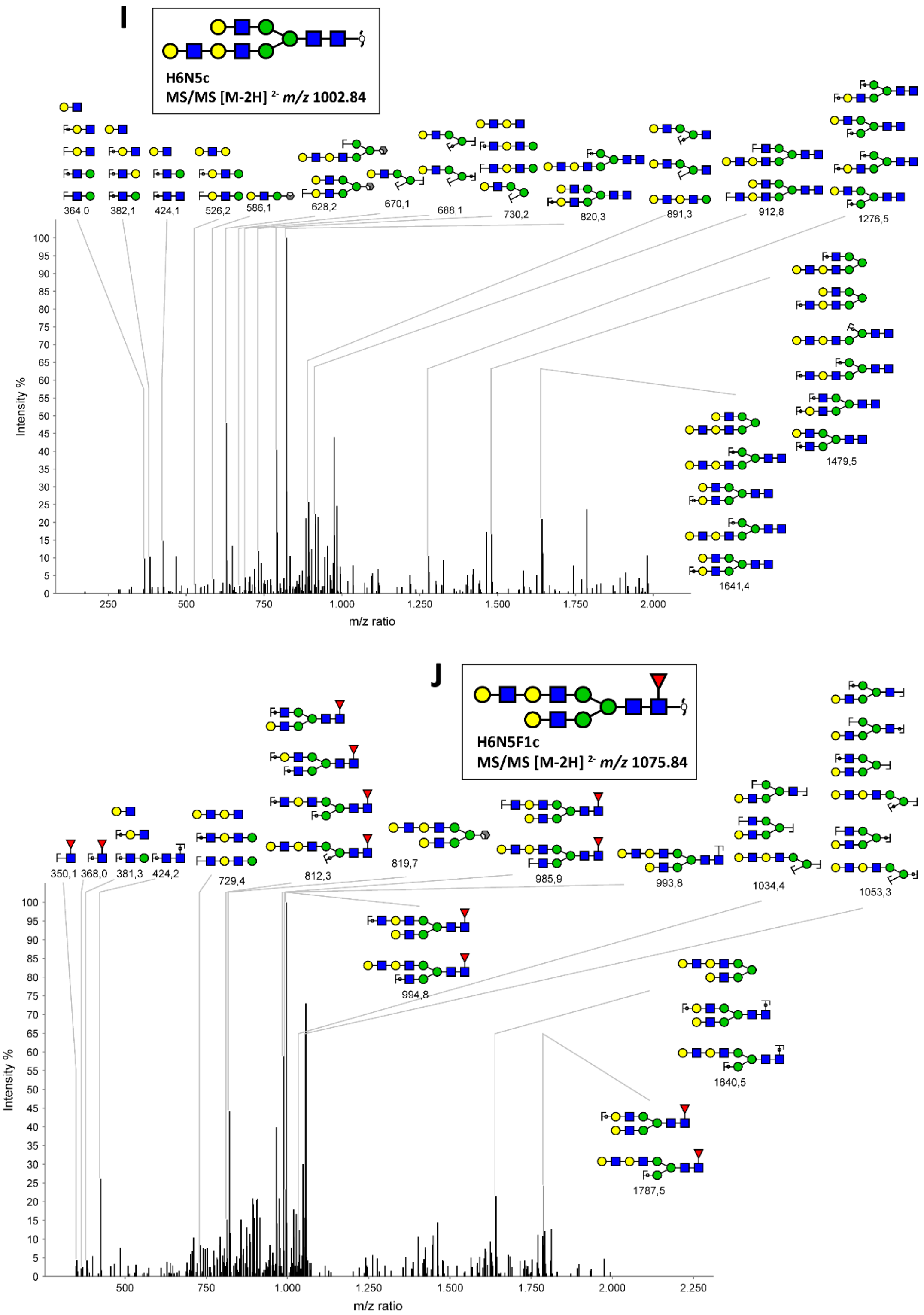

Supplementary Information Figure S4. MS/MS spectra of the selected *N*-glycans (*continued*): I) Repeated LacNAc carried *N*-glycan with composition H6N5c; **J)** Repeated LacNAc carried *N*-glycan with composition H6N5F1c. Blue square: *N*-acetylglucosamine, green circle: mannose, yellow circle: galactose, red triangle: fucose.

International Journal of Molecular Sciences

MDPI

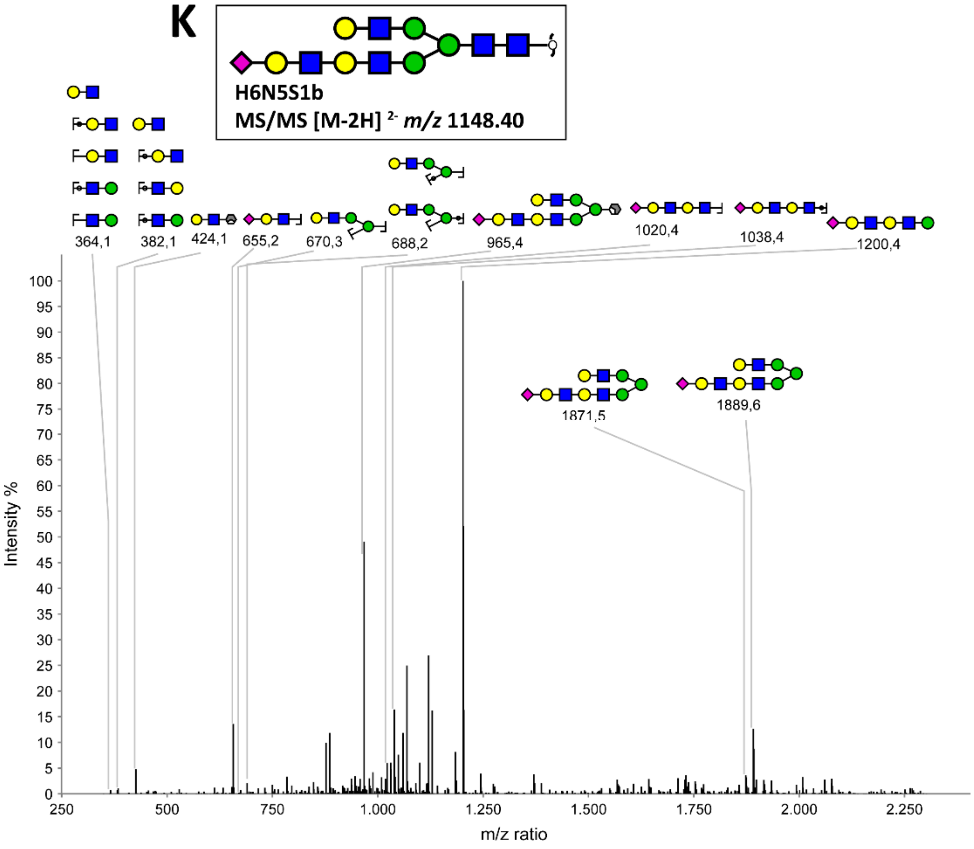

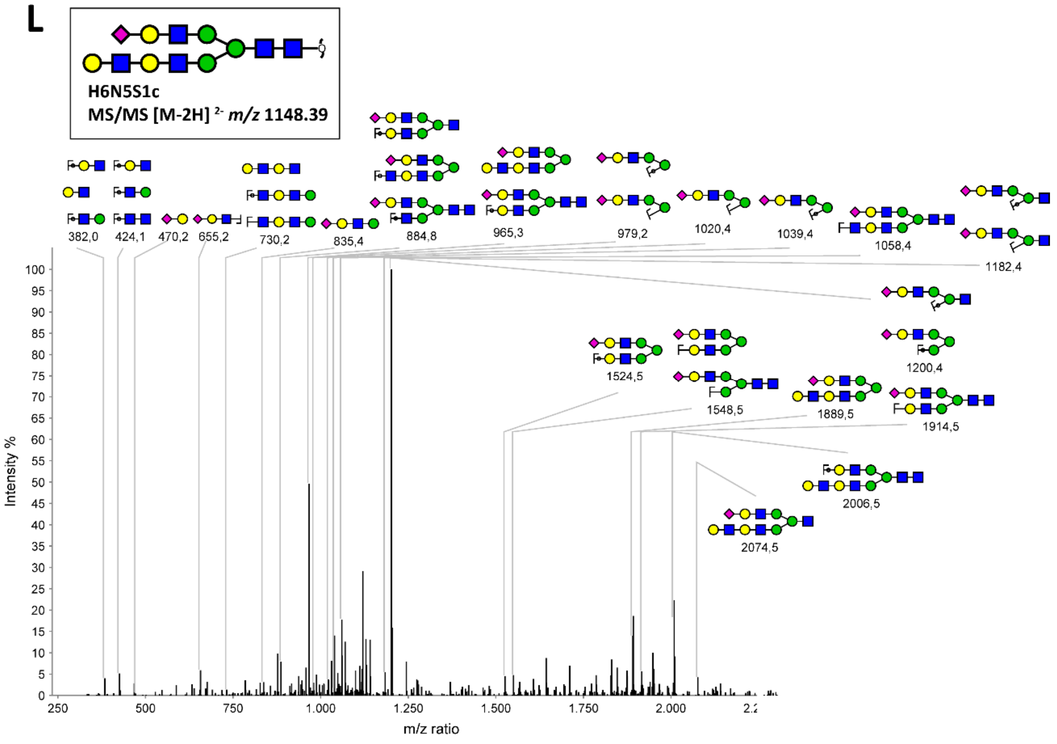

Supplementary Information Figure S4. MS/MS spectra of the selected *N*-glycans (*continued*): K) Repeated LacNAc carried *N*-glycan with composition H6N5S1b; **L)** Repeated LacNAc carried *N*-glycan with composition H6N5S1c. Blue square: *N*-acetylglucosamine, green circle: mannose, yellow circle: galactose, red triangle: fucose, purple diamond: *N*-acetylneuraminic acid.

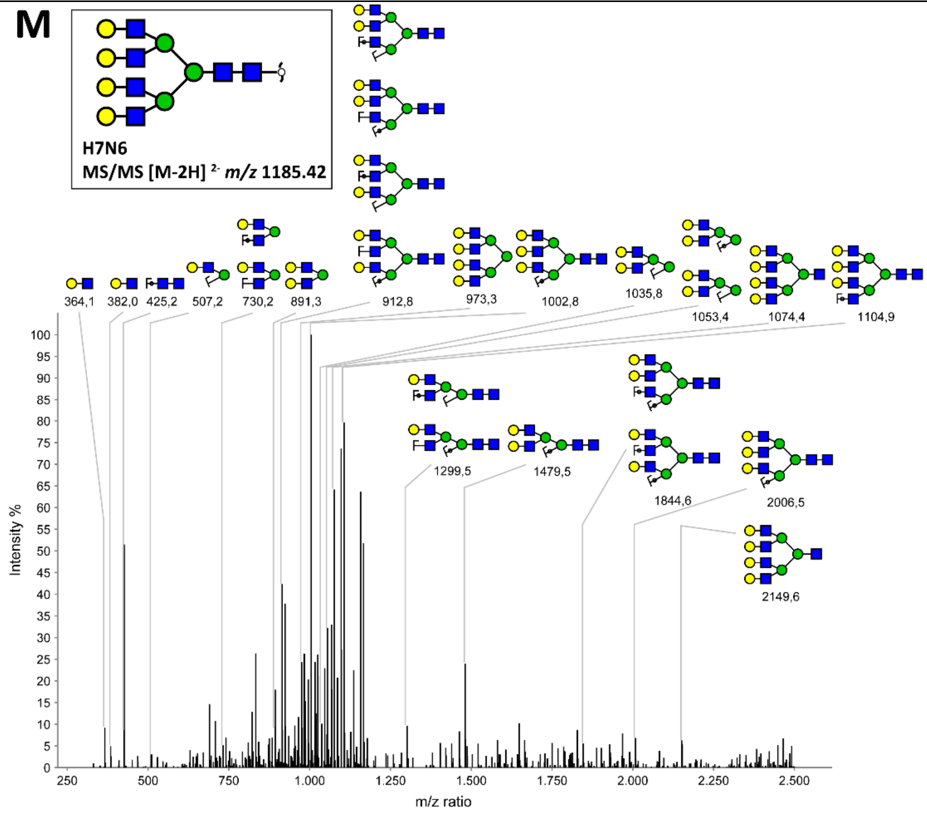

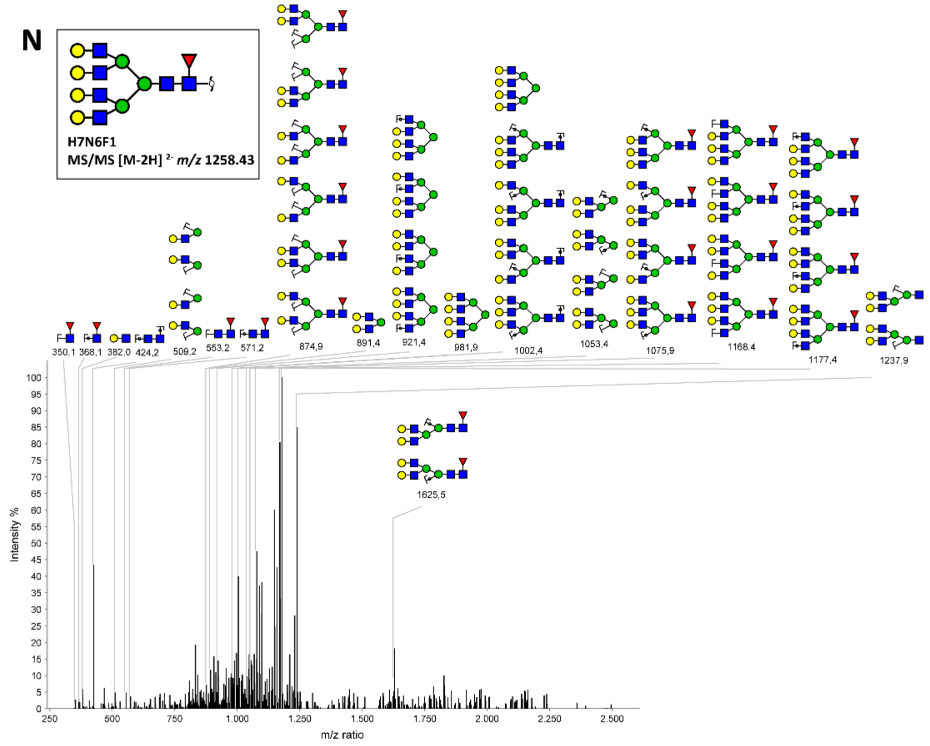

Supplementary Information Figure S4. MS/MS spectra of the selected *N*-glycans: **M)** tetraantennary *N*-glycan with composition H7N6. **N)** tetraantennary *N*-glycan with composition H7N6F1. Blue square: *N*-acetylglucosamine, green circle: mannose, yellow circle: galactose, red triangle: fucose.

International Journal of Molecular Sciences

Supplementary Information Figure S5

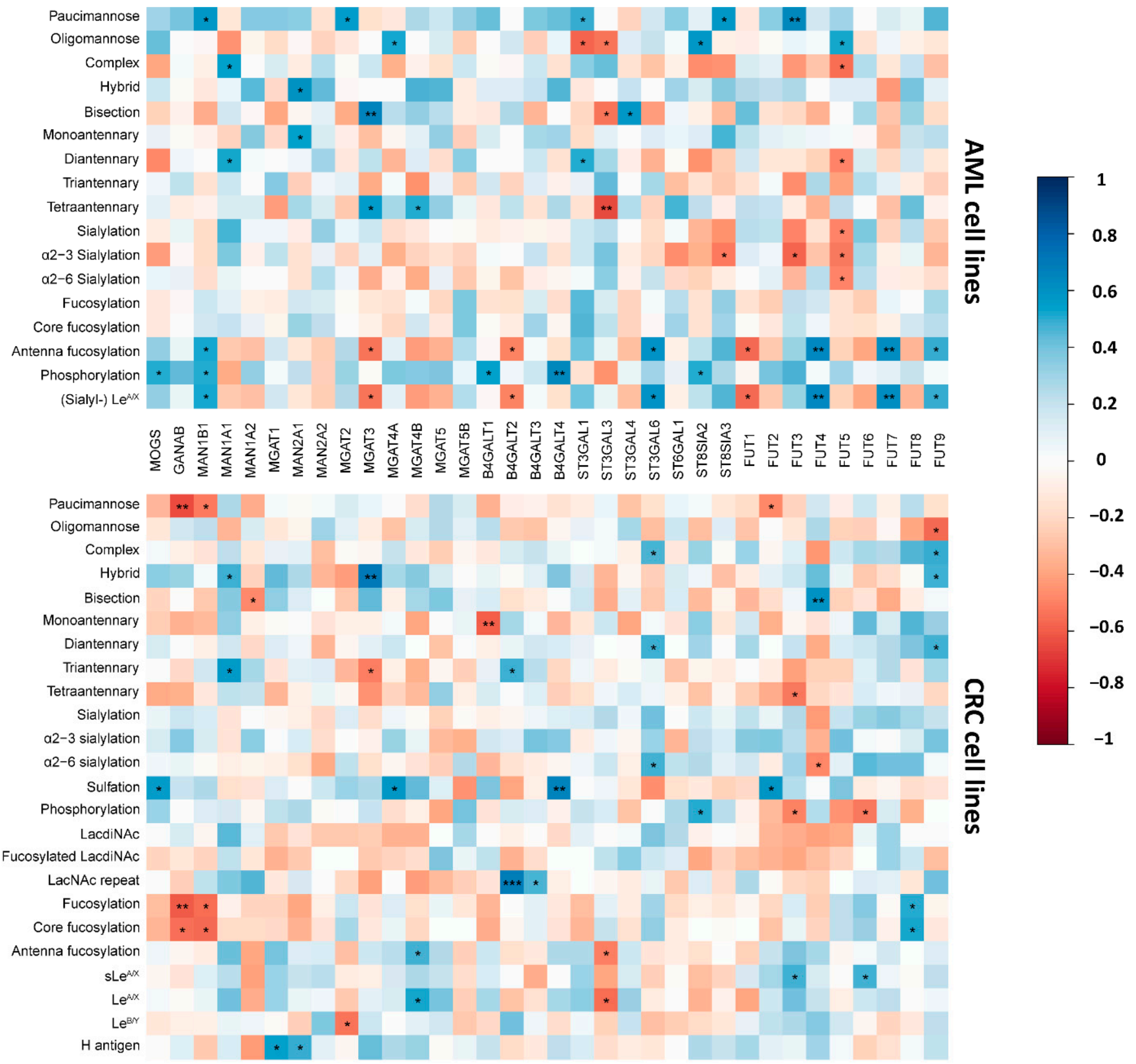

Supplementary Information Figure S5. Correlations of glycosylation with relevant GTs and differences of glycosylation features in CRC [1] and AML cell lines [2]. Correlation analysis was conducted between glycosylation features (relative quantification %) and corresponding GTs in CRC cell lines with the Spearman method.

International Journal of
Molecular Sciences
MDPI

Supplementary Information Figure S6

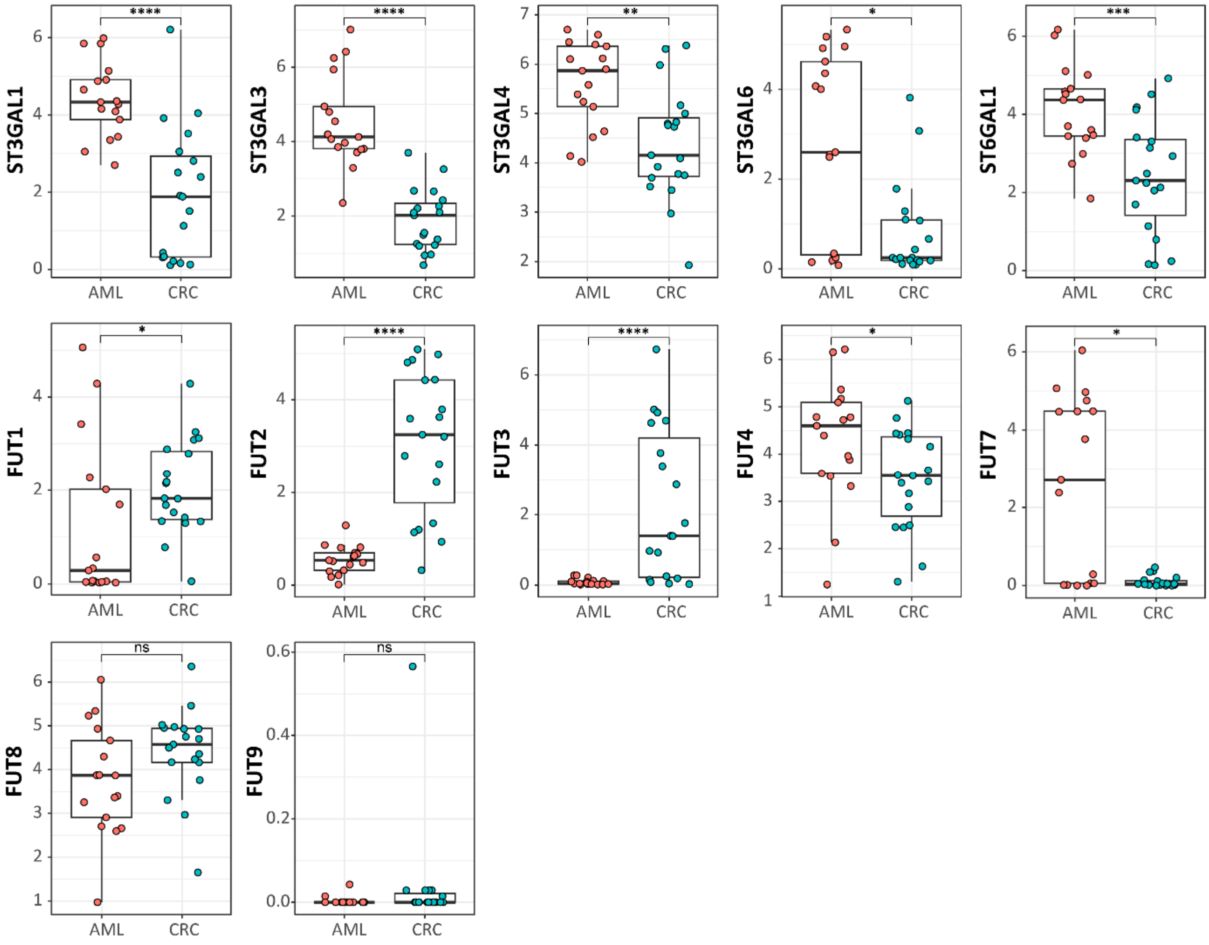

Supplementary Information Figure S6. The difference of transcriptomic expression levels of GTs between AML and CRC cell lines. The mRNA transcriptomics data of GTs of AML and CRC cell lines was extracted from a public dataset.[2] The difference were tested with Wilcoxon-Mann-Whitney non-parametric statistical test. P-values were corrected by the Benjamini-Hochberg method. Significant values are marked with * ($p \leqslant 0.05$), ** ($p \leqslant 0.01$), *** ($p \leq 0.001$) and **** ($p \leq 0.0001$). ns: not significant.

Supplementary Information Figure S7

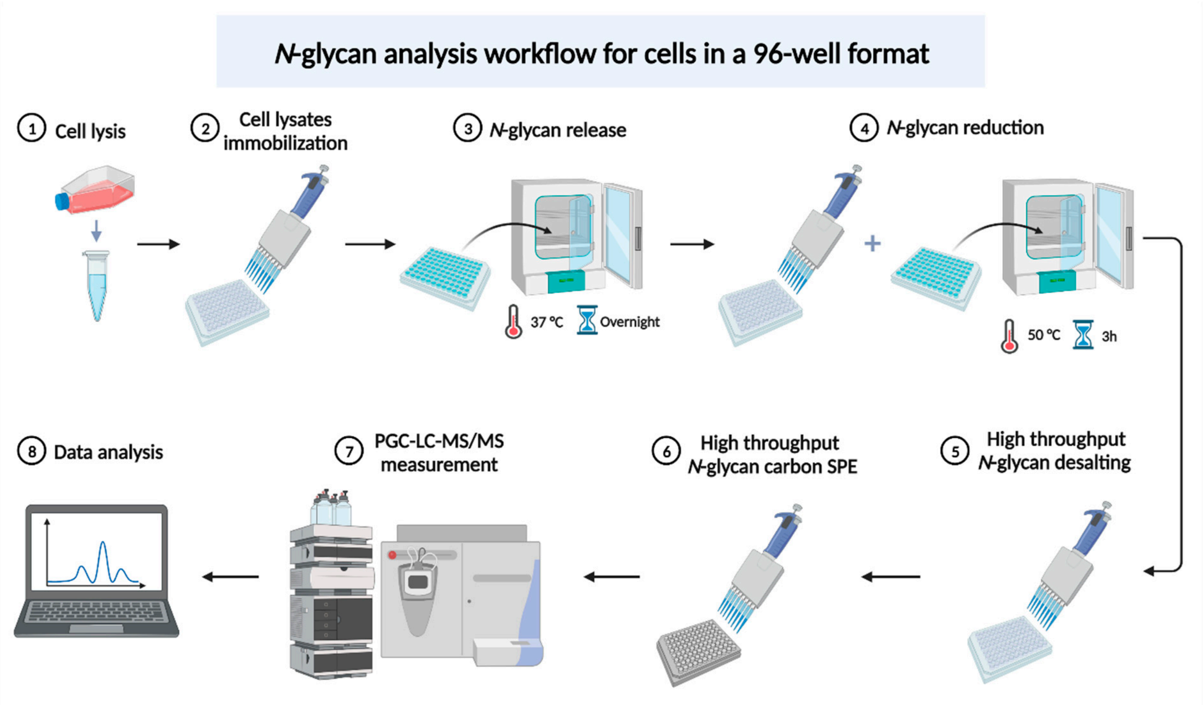

Supplementary Information Figure S7. *N*-glycan analysis workflow for cells in a 96-well format. (Created with BioRender.com)

References

1. Berg, K.C.G., et al., *Multi-omics of 34 colorectal cancer cell lines - a resource for biomedical studies.* Mol Cancer, 2017. **16**(1): p. 116.
2. Tsherniak, A., et al., *Defining a Cancer Dependency Map.* Cell, 2017. **170**(3): p. 564-576 e16.
